# Development and validation of the missed intensive nursing care scale

**DOI:** 10.1186/s12912-024-01805-3

**Published:** 2024-03-07

**Authors:** Li Yang, Wen Zhou, Yan Gao, Taiqin Wu, Huan Zhang, Xiuni Gan

**Affiliations:** Nursing Department, The Second Affiliated Hospital of Chongqing Medical University, Nanan District, Chongqing, China

**Keywords:** Missed nursing care, Unfinished nursing care, ICU, Scale development, Classical test theory, Item response theory

## Abstract

**Background:**

Missed nursing care is a pervasive issue in hospitals, nursing homes, and communities, posing a significant threat to patient safety and the quality of nursing care. It has adverse effects on patient satisfaction and the motivation of nursing staff. Understanding the causes and nature of these care omissions in clinical settings is essential for implementing effective interventions. This study aims to develop and validate a tool for assessing missed nursing care in adult intensive care units.

**Methods:**

Semi-structured interviews, expert consultations conducted via the Delphi method and item analysis were used to develop the initial scale. Our analysis involved data collected from 400 nurses and employed correlation coefficient analysis, critical ratio assessment, Cronbach’s α coefficient evaluation, discrete trend analysis, and factor analysis, which were grounded in both classical test theory and item response theory, allowing us to scrutinize and refine the items in the scale. To validate the scale, we conveniently sampled 550 nurses and assessed structural validity, internal reliability, split-half reliability, and test-retest reliability to ensure the scale’s robustness and accuracy.

**Results:**

The Missed Intensive Nursing Care Scale (MINCS) comprises three distinct components. Part A serves to collect general information about the participants. In Part B, the missed care elements are categorized into five domains, following the framework of Maslow’s hierarchy of needs theory: physiology, safety, belongingness, esteem, and cognition. Part C is dedicated to detailing the reasons behind missed care, which encompass labor resources, material resources, communication factors, and managerial factors. Remarkably, the Cronbach’s α coefficient for the MINCS stands at an impressive 0.951, with S-CVI values of 0.988 and 0.977 in Part B and C, respectively, underscoring the scale’s exceptional reliability and validity. This demonstrates the scale’s effectiveness in measuring missed nursing care while upholding rigorous standards of quality.

**Conclusions:**

The MINCS emerges as a robust and dependable instrument for quantifying instances of missed care within the ICU. Its efficacy makes it a valuable resource for informing the development of strategies aimed at averting and mitigating the adverse effects associated with missed nursing care.

**Supplementary Information:**

The online version contains supplementary material available at 10.1186/s12912-024-01805-3.

## Background

Missed Nursing Care (MNC), a phenomenon defined as “any aspect of the required patient care that is omitted partially or as a whole, or delayed remarkably” [1] has garnered significant attention in the realms of patient safety and nursing care quality. It is also referred to using various terms such as implicit rationing of nursing care, task undone, care left undone, unmet nursing care needs and unfinished nursing care [2]. “Required patient care” is a broad concept, encompassing care that should adhere to professional standards and include clinical, emotional, and administrative nursing care [3]. When required patient care is omitted, it can lead to adverse consequences for patient safety and the quality of nursing care services.

Associations between missed nursing care and adverse patient outcomes (e.g. infections, falls, long hospitalization, and increased mortality) have been explored [4]. As an element of the “structure-process-outcome” model, MNC affects the process of delivering nursing activities and finally decreases the quality of all nursing care services [5]. As a result, measuring MNC helps nurse managers find the potential risk that influences patient safety and prevents a worse situation from occurring.

Numerous studies have explored methodologies for evaluating MNC, encompassing approaches like nurses’ self-reported scales, direct observation methods [6], and retrospective chart review [7]. Presently, the predominant method is the use of nurses’ self-reported scales [8]. In terms of nurse-reported forms, many rely on inventories of tasks rooted in nursing duties [9]. Specifically, these instruments have been crafted from the standpoint of required nursing activities rather than focusing on patient needs. Under this idea, measurement tools for assessing MNC can be summarized in three generations [10]. The first generation comprises family instruments such as the MISSCARE survey [11], the Basel Extent of Rationing of Nursing Care (BERNCA) [12], and the Task Undone questionnaire [13]. The second generation primarily involves the validation of these three families of tools in different cultural and linguistic contexts while maintaining their original formats [14, 15]. The third generation consolidates the commonalities among these three families of tools and creates a unified measurement survey, facilitating the comparison of research results and illustrating the evolution of nursing service models [16]. In our view, there is also a fourth generation of tools designed to measure MNC in various specialized care units catering to distinct patient populations, such as neonatal intensive care units [17], pediatrics [18], nursing homes [19], maternity care settings [20], operating rooms [21], and oncology units [22]. However, as of now, there is no measurement tool specifically tailored for detecting MNC in adult intensive care units (ICUs). Patients admitted to the ICU are critically ill, and the quality of nursing care plays a pivotal role in their prognosis. Evidence suggests that omitted nursing care exists in ICUs and is associated with patient outcomes [23]. Given the unique and highly specialized nature of necessary nursing care activities provided to ICU patients, the development of a specialized instrument is warranted to identify any missed nursing activities and their underlying reasons in this setting.

Conversely, all the aforementioned tools share similar items when describing missed interventions, with these interventions being considered independent activities [11]. Consequently, excessive focus on nursing tasks has marginalized patient-centered ideas, leading that not all the needs of patients are valued and met [5]. Drawing from the principle of the Fundamental of Care (FoC) framework, only the Perceived Implicit Rationing of Nursing Care (PIRNCA) and the Unfinished Care tool measure the relational dimension out of the three domains outlined in the framework [24]. How the care plan is formulated depends on the patient’s needs, and the care should address their physiological, psychological, and social needs [25].

Furthermore, it is worth noting that the psychometric properties of these scales are currently being assessed. Among the most studies, the structural validity has remained untested because the part of missed elements is commonly viewed as unidimensional [10]. Likewise, classical test theory (CTT) was chosen frequently, item response theory (IRT), a modern measurement theory, has rarely been utilized in development and validation research within this field. Notably, Bassi et al. utilized Mokken analysis to establish the unidimensionality of the unfinished nursing care (UNC) tool [10], while Riklikiene et al. employed Rasch analysis in their validation study of the Infection Prevention and Control Survey [26]. These approaches offer a fresh perspective for the development and evaluation of measurement tools in this domain.

Hence, even though research in this field is ongoing, there remains a need for further investigation into MNC within specialized contexts. A new perspective and multidimensional approach should be considered when designing elements of missed interventions to ensure a comprehensive understanding of nursing care. This study aimed to develop and appraise the psychometric properties of the missed intensive nursing care scale (MINCS) using both CTT and IRT, thus enhancing the body of knowledge regarding ICU-specific research and furnishing valuable measurement tools aimed at enhancing the quality of care in the intensive care unit (ICU).

## Methods

### Design

**Fig. 1:**
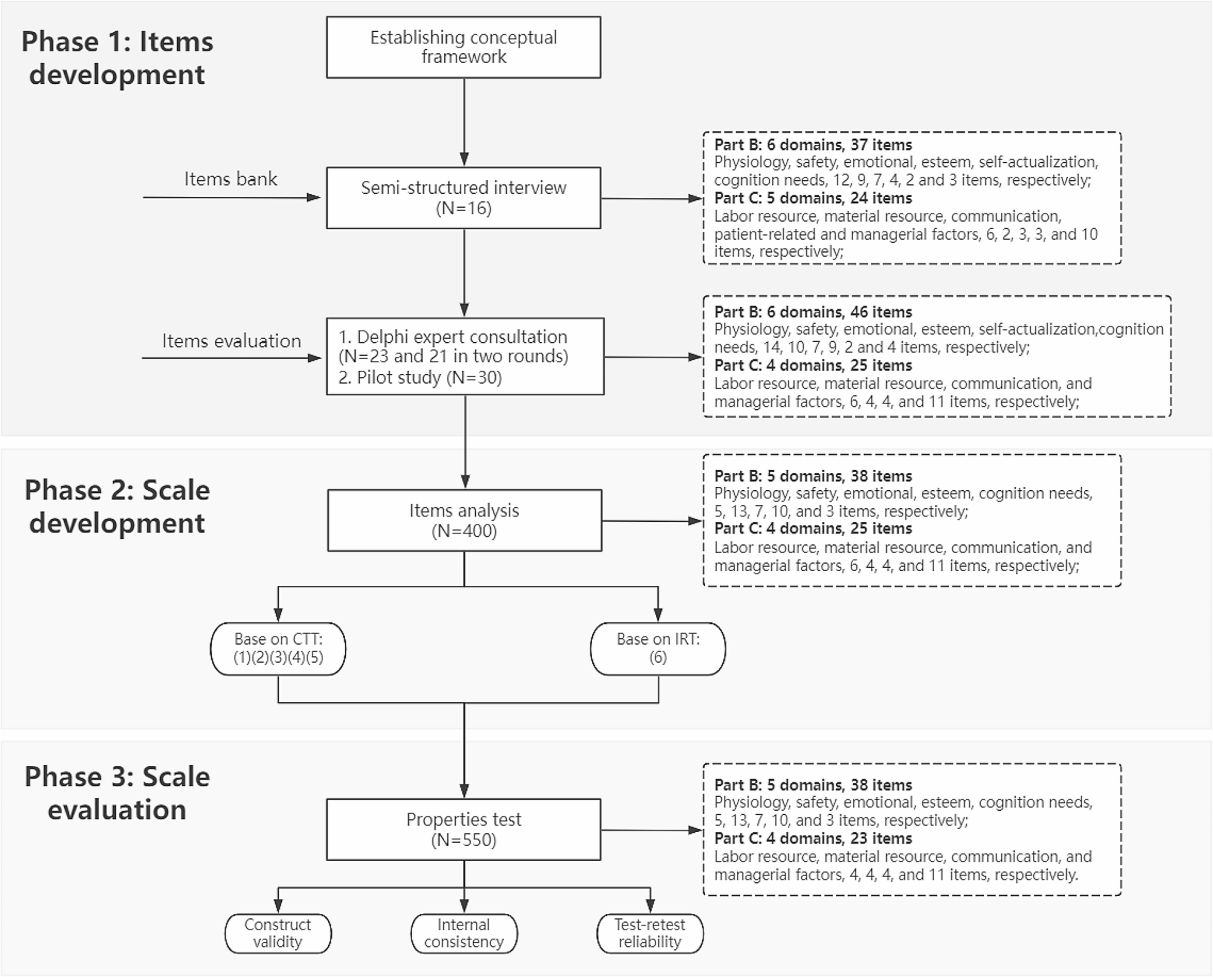
The development procedure of the missed intensive nursing care scale. *Note.* (1) represents correlation coefficient analysis; (2): The critical ratio; (3): Cronbach’s α coefficient analysis; (4): Discrete tendency analysis; (5): Factor analysis; (6) Evaluation of the discrimination and difficulty parameters

An instrument development and validation study design was adopted following the three phases between June 2022 and October 2023: (1) Phase 1: Items development; (2) Phase 2: Scale development; (3) Phase 3: Scale evaluation. A flow chart depicted the whole process (Fig. 1).

### Phase 1: items development

**Establish theoretical framework** The MISSCARE survey [11], developed by Kalisch, primarily comprises two key components: one for identifying missed nursing activities or elements, and another for uncovering potential reasons for such missed care behavior. For the development of the MINCS, we closely followed the format that Kalisch used, which essentially consists of three integral parts: Part A, dedicated to capturing essential demographic information of the respondents; Part B, featuring items related to missed intensive nursing activities; and Part C, focusing on elucidating the reasons behind missed care behaviors. After an extensive search of the literature, we chose to adopt Maslow’s hierarchy of needs theory [27] as the theoretical framework in Part B. It is worth noting that prior research [5, 24] has pointed out that existing tools for measuring missed care predominantly emphasize patients’ physical needs while neglecting their psychological and social needs. While nursing services should meet people’s physiological, social, psychological, and spiritual needs based on individual’s belief and preference [28]. Maslow’s theory categorizes human needs into seven key aspects: physiological, safety, belongingness, esteem, self-actualization, cognitive, and aesthetic needs. There were studies [29, 30] employing Maslow’s theory to explore the care needs of critically ill patients in ICU settings, revealing that a significant proportion of these needs often remain unmet. This, in turn, results in suboptimal nursing outcomes and a diminished patient experience. Hence, we have selected this theory with the aim of identifying potential shortcomings in the caring process, gaining a comprehensive understanding of which aspects of patients’ needs are inadequately addressed, and ultimately facilitating the implementation of patient-centered holistic care by addressing these underlying issues.

**Generating the item bank** To generate the items for the MINCS, a research team was initially formed, consisting of a team of six members, including a nursing professor, two nursing doctoral candidates, and three nursing master’s students. Subsequently, semi-structured interviews were conducted, 16 ICU nurses of varying gender, age, educational level, working experience, job title, and facilities were recruited by purposeful sampling from 7 tertiary hospitals across 4 provinces in China. These interviews were guided by Maslow’s hierarchy of needs theory. The interviews outline is shown in Appendix S1. During the interviews, participants were asked how they meet these needs of patients, whether there were instances of missed nursing care during this process, and the reasons for such missed care, and then we developed items for each dimension based on their responses. The method of thematic analysis was utilized to analyze the gathered data. In addition to the insights gained from the interviews, we referred to professional standards and regulations that offer specific guidance to nurses on the care they should provide to critically ill patients. These standards and regulations helped us summarize the core competencies and skills that an ICU nurse should possess. This enriched the content of our research, ensuring a comprehensive and well-informed approach to item generation for the MINCS. For Part C, we referred to the scale developed by Kalisch and made some revisions based on the interview results. Finally, 37 items in Part B and 24 items in Part C were initially formed.

#### Items evaluation

A preliminary evaluation and revision towards the items were achieved by Delphi expert consultation and a pilot test.

The experts were purposively sampled based on the following criteria: (1) had a minimum of 10 years of experience working in the ICU; (2) specialized in clinical nursing, nursing management, or education; and (3) possessed at least an undergraduate degree. Resulting in the participation of 23 and 21 individuals in the first and second rounds, respectively. These experts were from 12 different provinces in China and requested to assess each item in the scale for its importance and relevance, using a rating scale that ranged from 1 (least important) to 5 (most important). The item content validity index (I-CVI) for each item was determined by the proportion of experts rating it as 3 or 4, divided by the total number of experts. The scale content validity index (S-CVI), representing the overall content validity of the scale, was computed as the average of all item I-CVI scores. The retention standard is above 0.80 [31]. Subsequently, the scale items were refined based on the suggestions provided by the panel of experts, leading to an expanded set of 47 items for Part B and 25 items for Part C. Any items with an average rating score of < 3.5 and a coefficient of variation (CV) ≥ 0.25 were removed from the scale. This rigorous process ensured that only the most pertinent and valuable items were retained for the MINCS.

Surface validity was also assessed by the abovementioned experts, their opinions on the appropriateness, suitability and practicality of the scale were gathered, and we made some adjustments according to their feedback.

A pilot study was carried out to evaluate the readability and comprehensibility of the items included in the MINCS. This pilot study involved 30 nurses from the ICU of the researcher’s hospital. Based on the feedback received through face-to-face inquiry with participants, it was identified that certain items in Part B of the MINCS had redundant expressions with similar meanings. Consequently, these duplicate items were consolidated into a single item, streamlining and improving the overall clarity and conciseness of the scale. Until this stage, there were 46 items in Part B and 25 items in Part C.

### Phase 2: scale development

#### Items analysis

The items were analyzed and screened under the guidance of CTT and IRT.

**Classical test theory (CTT)** There were five methods used to screen the items: (1) correlation coefficient analysis: the correlation coefficient (r) between an item and the total score ≥ 0.4 was retained [32]. (2) The critical ratio analysis: the total scores of the scale were ranked from high to low, with the top 27% and the bottom 27%. Items with a decision value (t value) greater than 3, and a statistically significant difference between the two groups (*p* < 0.05), were retained. (3) Cronbach’s α coefficient analysis: Items that, when removed, resulted in an increase in the Cronbach’s α coefficient were considered for potential removal. (4) Discrete tendency analysis: This method assesses the sensitivity of items. Items with a larger standard deviation (SD) were indicative of stronger discriminative ability among respondents. The SD of each item’s scores was calculated, and items with an SD of less than 0.7 were considered for removal [33]. (5) Factor analysis: An exploratory factor analysis (EFA) was conducted, and the ‘Principal Component’ method was used for factor extraction. Items with factor loadings of ≥ 0.4 were retained following maximum variance rotation. However, items with similar load indices (with a difference of less than 0.1) in two or more factors without specificity were eliminated [34]. Prior to EFA, we calculated the Kaiser–Meyer–Olkin (KMO) measure (optimal value: >0.6) and performed the Bartlett test of sphericity (optimal value: *p* < 0.01) to confirm the suitability of the data for factor analysis [35].

**Item response theory (IRT)** Item Response Theory (IRT) is a method that seeks to elucidate the relationship between subjects’ responses to items and their underlying latent traits, addressing several of the limitations of CTT [36]. A fundamental assumption of IRT is unidimensionality, implying that the entire scale or each subscale measures only one latent trait. To assess unidimensionality, we conducted an exploratory factor analysis (EFA). A convincing indicator of unidimensionality was achieved if the ratio of the eigenvalues of the first and second unrotated EFA components exceeded 3:1 [37]. In our IRT analysis, we employed Samejima’s two-parameter graded response model (GRM) [38]. This model was chosen because it accommodates ordered polytomous items within our scale. We estimated both the discrimination parameter (a) and the difficulty parameter (b) for each item. Items with an ‘a’ value less than 0.7 and a ‘b’ value outside the range of -4.0 to 4.0 were considered for potential removal [39].

In this study, items that did not meet the criteria outlined in (5) were removed. Additionally, items that were recommended for removal by two or more methods within the remaining criteria (1), (2), (3), and (4) in CTT and IRT.

### Phase 3: scale evaluation

#### (1) Validity

**Structural validity** It was calculated by confirmatory factor analysis (CFA). The goodness-of-fit of the CFA models was evaluated utilizing the following indices and cutoff levels: the χ^2^-test/degrees of freedom ratio (χ^2^/df), root mean square error of approximation (RMSEA), standardized root mean square residual (SRMR), incremental fit index (IFI), Tucker–Lewis index (TLI), and comparative fit index (CFI). The fit was considered to be acceptable when the χ^2^/df < 5, RMSEA and SRMR were ≤ 0.08, and the IFI, TLI, CFI were ≥ 0.90 [40].

#### (2) Reliability

The reliability was validated using Cronbach’s α coefficient, split-half reliability and test–retest reliability. The Cronbach’s α coefficient of the total scale and each dimension were calculated, and *α* > 0.7 was acceptable reliability [41]. Split-half reliability was assessed by calculating the correlation between two halves of the scale, which were divided based on odd-numbered and even-numbered items [42]. A Guttman Split-half Coefficient exceeding 0.6 was considered acceptable [43]. The test-retest reliability was evaluated approximately 2 weeks after the initial test, and intraclass correlation coefficients (ICCs) were calculated to assess the stability and consistency of the scale over time.

### Participants

Participants were recruited from fourteen provinces and cities in mainland China using a convenience sampling approach. The inclusion criteria for the study were as follows: (1) registered nurses actively employed in adult intensive care units (ICUs), and (2) a voluntary willingness to participate in the study. Exclusion criteria applied to nurse students and trainee nurses. The sample was collected in two periods by online survey distribution, one for item analysis and exploratory factor analysis (EFA), and another for confirmatory factor analysis (CFA). Based on the standards of 5 to 10 times the number of scale items [44], and considering 10% invalid answers, the required sample size was determined to be a minimum of 390 cases. The questionnaire was distributed electronically using Questionnaire Star, an online crowdsourcing platform widely utilized in China.

### Data analysis

For the factor analysis, we utilized IBM SPSS Statistics version 25.0 and Amos version 25.0. Item screening was achieved through R 4.3.1.(irt GUI package) [45].

## Results

### General information of the participants

A total of 1000 ICU nurses participated in this study, and 950 valid samples were included. Among them, 796 were female (83.8%) and 154 were male (16.4%) with an average age of 31. The majority of them held a bachelor’s degree (818, 86.1%), and had worked in the ICU for at least 6 to 19 years (55.7%). A total of 526 individuals (55.4) engaged in critical nursing care in the comprehensive ICU, and the other top three specialty ICUs were 109 in the emergency ICU (11.5%), 98 in the surgical ICU (10.3%) and 71 in the medical ICU (7.5%). This diversity contributes to the overall representativeness of our sample, facilitating the generalizability of our study outcomes. A descriptive analysis of the participants’ demographic characteristics is listed in Table 1.

**Table 1:** The basic characteristics of participants

Characteristics	N(%)	Characteristics	N(%)
**Gender**		**Hospital level**	
Male	154(16.2)	Tertiary hospital	867(91.3)
Female	796(83.3)	Secondary hospital	83(8.7)
**Age (years)**		**Work department**	
20 ~ 30	479(50.4)	GICU	526(55.4)
31 ~ 40	409(43.1)	MICU	71(7.5)
41 ~ 50	59(6.2)	SICU	98(10.3)
>50	3(0.3)	EICU	109(11.5)
**Marital status**		RICU	60(6.3)
Single	597(62.8)	NICU	69(7.3)
Married	341(35.9)	Others	17(1.8)
Others	12(1.3)	**ICU specialist**	
**Education level**		Yes	285(30)
Diploma/associate’s degree	109(11.5)	No	665(70)
Bachelor’s degree	818(86.1)	**Average working** **hours per week**	
Master’s degree and higher	23(2.4)	≤ 40 h	394(41.5)
**Job titles**		41 ~ 48 h	449(47.3)
Nurse	172(18.1)	>48 h	107(11.3)
Nurse practitioner	410(43.2)	**Working shift**	
Nurse-in-charge	331(34.8)	8 h	599(63.1)
Associate director	33(3.5)	12 h	335(35.3)
Director of nurses	4(0.4)	Others	16(1.7)
**Working position**		**Average number of** **care patients per shift**	
Staff nurse	897(94.4)	1 ~ 2	164(17.3)
Head nurse	53(5.6)	3 ~ 4	674(70.9)
**Years working in ICU**		More than 5	112(11.8)
≤ 1	91(9.6)		
1 ~ 5	289(30.4)		
6 ~ 10	297(31.3)		
11 ~ 19	232(24.4)		
≥ 20	41(4.3)		

*Abbreviations:* GICU, general intensive care unit; MICU, medical intensive care unit; SICU, surgical intensive care unit; EICU, emergency intensive care unit; RICU, respiratory intensive care unit; NICU, neurological intensive care unit

### Feasibility

The time frame was restricted in the last shift to decrease respondents’ recall burden and all of the participants completed the questionnaire in a mean time of six minutes and without any items being omitted in three parts of the scale. This indicates that our scale has a high level of acceptability.

### Results of items development

Informed by a comprehensive literature review and semi-structured interviews, we constructed an item bank comprising 37 items in Part B and 24 items in Part C of the MINCS. From the perspective of the interviewees, meeting the aesthetic needs of patients in the ICU primarily involves maintaining their physical cleanliness and comfort, which is considered part of fundamental care. As a result, these items were more appropriately categorized under the physiological needs domain. Consequently, the items in Part B were divided into six distinct domains: nursing interventions related to satisfying patients’ physiological needs, safety needs, emotional needs, esteem needs, self-actualization needs, and cognitive needs. For scoring, participants were asked to rate the items in Part B on a five-point Likert scale, with options ranging from 1 (never missed) to 5 (always missed), and an extra choice for “not applicable (NA)”. A higher score indicated a higher frequency of missed care for a given item. Items in Part C pertained to five domains: labor resources, material resources, communication, patient-related factors and managerial factors. These items were rated on a four-point Likert scale, where factors were assessed as significant, moderate, minor, or not a reason for missed care, assigned scores of 4, 3, 2, and 1 points, respectively. A higher score for an item in Part C indicated a greater relevance to missed care.

A total of 23 and 21 experts participated in two rounds of consultation. It is noteworthy that the response rates of experts in both rounds were high, with rates of 92% and 91%, respectively. This high level of expert engagement underscores the significance and positive participation in the study. Furthermore, the Kendall coordination coefficient *W* was calculated and found to be 0.133 and 0.141 for the two rounds of consultation, respectively. In both cases, the p value was less than 0.001. During these consultations and discussions with team members, modifications were made to the structure of the MINCS, enhancing its quality and relevance.

A preliminary version of the scale after pilot test, denoted as MINCS-v1, was created. This version consisted of 46 items in Part B and 25 items in Part C. The S-CVI for Part B and Part C was impressively high, with values of 0.988 and 0.977, respectively. Additionally, the item I-CVI in Part B ranged from 0.952 to 1, while in Part C, it ranged from 0.857 to 1. These high CVI values underscore the content validity and robustness of the scale, indicating that it effectively measures the construct of missed intensive nursing care.

### Results of scale development

A comprehensive set of item screening methods was employed, including the correlation coefficient method, critical ratio analysis, Cronbach’s α coefficient, discrete tendency analysis, factor analysis, and item response theory. Items were rigorously assessed and discarded based on stringent criteria. The Kaiser‒Meyer‒Olkin (KMO) measure yielded a high value of 0.972 in Part B and 0.963 in Part C, and Bartlett’s test of sphericity was both significant (*p* < 0.01), confirming the appropriateness of the data for factor analysis. Principal component analysis revealed that the ratio of the eigenvalues of the first and second unrotated EFA components exceeded 3 for all dimensions in Part B and Part C, indicating the unidimensional nature of each factor. These factors collectively accounted for 76.9% of the variance in Part B and 75.42% in Part C (Table 2). As a result, several items were removed during the screening process: Part B: Items B1-4, B1-8, B1-9, B1-12, B1-13, B1-14, B4-9, and B6-1 were deleted due to similar load coefficients in two or more factors. Part C: There were four items under the domain of communication showing cross-loadings on two factors and should have been deleted, however, considering that communication is a crucial contributor to missed nursing care [46], we ultimately decided to retain them. Additionally, for items B2-4, B3-2, B4-3, B4-7, and B4-9, none of the respondents selected the fifth category, leading to the analysis of these items using only four categories. The results are shown in Table 3. Furthermore, it was noted that in Part B, the domain of nursing activities related to satisfying self-actualization needs had only two items. This limitation precluded the calculation of Cronbach’s α. During interviews, respondents had different opinions on whether ICU patients had self-actualization needs. Consulting relevant experts, it was eventually concluded that patients do indeed have such needs, which can be fulfilled with the assistance of medical staff, provided that full respect is given to the patients. Consequently, the two items were merged into the domain of esteem needs, considering the real situation of critically ill patients.

**Table 2:** Results of the item selection in Part B and Part C using CTT and IRT

	CTT					IRT					Final outcome
Item	(1)	(2)	(3)	(4)	(5)	a	b1	b2	b3	b4	
**B1-1**	0.715	13.724	0.952	0.82	0.617	2.679	0.105	1.208	1.938	3.019	**√**
**B1-2**	0.752	13.808	0.951	0.75	0.578	3.015	0.206	1.307	2.036	3.193	**√**
**B1-3**	0.790	17.901	0.950	0.99	0.689	2.665	-0.398	0.725	1.577	2.536	**√**
**B1-4**	0.821	18.080	0.949	0.82	0.579*	3.306	0.029	1.077	1.786	3.098	**×**
**B1-5**	0.782	18.375	0.951	1.07	0.732	2.392	-0.436	0.759	1.403	2.355	**√**
**B1-6**	0.768	17.190	0.951	1.04	0.768	2.097	-0.737	0.568	1.495	2.525	**√**
**B1-7**	0.758	15.226	0.952	1.07	0.783	2.123	-0.62	0.624	1.433	2.57	**√**
**B1-8**	0.825	15.665	0.949	0.84	0.566*	2.783	-0.15	1.078	1.866	2.779	**×**
**B1-9**	0.823	18.017	0.949	0.85	0.527*	3.465	-0.12	1.036	1.734	3.09	**×**
**B1-10**	0.839	21.069	0.949	0.92	0.575	3.7	-0.231	0.86	1.555	2.494	**√**
**B1-11**	0.833	18.171	0.949	0.79	0.572	4.16	0.044	1.039	1.888	2.627	**√**
**B1-12**	0.870	21.024	0.948	0.84	0.485*	4.832	-0.076	0.883	1.724	2.903	**×**
**B1-13**	0.800	18.875	0.950	0.83	0.485*	3.848	0.009	1.039	1.794	2.492	**×**
**B1-14**	0.772	15.359	0.951	0.93	0.491*	3.172	-0.122	0.953	1.679	2.633	**×**
**B2-1**	0.823	11.851	0.956	**0.64**	0.772	3.592	0.606	1.504	2.208	3.041	**√**
**B2-2**	0.864	11.953	0.955	**0.67**	0.786	4.365	0.606	1.386	1.987	2.933	**√**
**B2-3**	0.788	14.587	0.961	0.91	0.554	3.379	0.22	1.103	1.746	2.277	**√**
**B2-4**	0.822	13.238	0.957	0.74	0.702	4.101	0.396	1.294	2.038	N/A	**√**
**B2-5**	0.871	14.369	0.954	0.73	0.771	3.766	0.369	1.338	1.987	2.674	**√**
**B2-6**	0.895	15.585	0.953	0.73	0.736	4.842	0.342	1.257	1.892	2.904	**√**
**B2-7**	0.903	18.914	0.953	0.70	0.729	5.272	0.282	1.21	2.068	2.874	**√**
**B2-8**	0.899	19.329	0.953	0.74	0.694	5.555	0.247	1.142	1.883	2.855	**√**
**B2-9**	0.860	15.432	0.955	0.73	0.649	5.133	0.37	1.255	1.825	2.879	**√**
**B2-10**	0.890	14.889	0.954	**0.67**	0.754	6.066	0.491	1.318	2.062	2.823	**√**
**B3-1**	0.818	17.901	0.950	1.08	0.663	2.705	-0.182	0.81	1.556	2.548	**√**
**B3-2**	0.856	19.612	0.942	0.79	0.608	3.884	0.019	1.051	1.816	N/A	**√**
**B3-3**	0.922	20.858	0.934	0.94	0.734	3.679	-0.047	0.903	1.567	2.203	**√**
**B3-4**	0.912	22.353	0.936	0.91	0.703	4.076	-0.055	0.889	1.648	2.283	**√**
**B3-5**	0.909	22.236	0.936	0.91	0.671	3.821	-0.129	0.847	1.615	2.27	**√**
**B3-6**	0.868	21.320	0.942	1.01	0.656	3.3	-0.215	0.849	1.437	2.086	**√**
**B3-7**	0.864	20.441	0.941	0.85	0.563	4.126	0.003	0.952	1.726	2.415	**√**
**B4-1**	0.842	16.132	0.944	0.80	0.633	4.116	0.32	1.181	1.804	2.971	**√**
**B4-2**	0.848	18.127	0.944	0.94	0.638	3.583	0.018	0.973	1.708	2.214	**√**
**B4-3**	0.865	16.003	0.943	0.75	0.649	4.957	0.293	1.113	1.829	N/A	**√**
**B4-4**	0.861	19.641	0.944	0.98	0.662	3.575	-0.02	0.898	1.509	2.495	**√**
**B4-5**	0.868	19.355	0.942	0.93	0.665	3.583	0.001	0.985	1.51	2.707	**√**
**B4-6**	0.893	21.234	0.941	0.81	0.648	4.971	0.088	0.986	1.749	2.502	**√**
**B4-7**	0.868	19.372	0.943	0.78	0.678	4.334	0.128	1.067	1.812	N/A	**√**
**B4-8**	0.848	19.581	0.945	0.98	0.673	4.33	0.037	0.918	1.615	2.568	**√**
**B4-9**	0.735	11.899	0.950	0.72	0.587*	3.423	0.44	1.313	2.015	N/A	**×**
**B5-1**	0.963	18.816	N/A	0.96	0.592	3.494	-0.093	0.945	1.579	2.334	**√**
**B5-2**	0.958	20.200	N/A	0.91	0.644	4.213	0.006	0.917	1.714	2.956	**√**
**B6-1**	0.805	14.417	0.892	0.81	0.535*	3.74	0.281	1.181	1.856	3.039	**×**
**B6-2**	0.896	16.107	0.845	0.89	0.579	3.868	0.211	1.097	1.792	2.47	**√**
**B6-3**	0.899	18.053	0.847	0.98	0.596	3.384	-0.065	0.943	1.615	2.337	**√**
**B6-4**	0.882	18.436	0.861	0.99	0.598	2.929	-0.062	0.963	1.617	2.376	**√**
**C1-1**	0.630	8.914	**0.892**	0.92	0.858	0.928	0.321	1.522	3.491		**√**
**C1-2**	0.698	10.610	**0.880**	0.94	0.741	1.137	-0.211	1.145	2.734		**√**
**C1-3**	0.849	17.836	0.847	0.97	0.689	2.043	-0.994	0.212	1.373		**√**
**C1-4**	0.846	18.095	0.848	1.00	0.832	2.152	-0.975	0.078	1.212		**√**
**C1-5**	0.841	19.164	0.849	0.98	0.765	2.18	-0.868	0.248	1.404		**√**
**C1-6**	0.873	21.503	0.841	1.01	0.804	2.389	-0.964	0.144	1.109		**√**
**C2-1**	0.907	21.905	0.884	0.99	0.771	2.278	-1.38	-0.057	0.801		**√**
**C2-2**	0.902	21.563	0.888	1.03	0.790	2.07	-1.279	-0.097	0.835		**√**
**C2-3**	0.904	21.999	0.886	1.01	0.788	2.139	-1.275	-0.051	0.87		**√**
**C2-4**	0.866	20.001	0.909	1.02	0.755	1.957	-1.482	-0.172	0.695		**√**
**C3-1**	0.905	25.563	0.929	1.02	**0.610**	3.386	-0.981	-0.042	0.808		**√**
**C3-2**	0.928	23.058	0.914	0.99	**0.541**	3.048	-0.94	-0.07	1.035		**√**
**C3-3**	0.931	21.936	0.912	0.97	**0.575**	3.398	-0.979	0.029	1.01		**√**
**C3-4**	0.912	21.855	0.922	0.94	**0.539**	3.138	-1.225	-0.096	0.927		**√**
**C4-1**	0.798	21.111	0.960	1.08	0.686	2.504	-0.775	0.082	0.953		**√**
**C4-2**	0.840	21.603	0.958	1.01	0.758	2.986	-1.184	-0.208	0.682		**√**
**C4-3**	0.837	26.062	0.958	1.03	0.678	3.562	-1.051	-0.16	0.624		**√**
**C4-4**	0.824	21.297	0.959	1.03	0.735	2.898	-1.058	-0.144	0.782		**√**
**C4-5**	0.862	26.905	0.957	1.03	0.746	3.698	-1.045	-0.137	0.679		**√**
**C4-6**	0.828	24.600	0.959	1.03	0.710	3.042	-1.067	-0.165	0.715		**√**
**C4-7**	0.887	31.547	0.956	1.02	0.740	4.805	-1.06	-0.192	0.537		**√**
**C4-8**	0.895	34.126	0.956	1.05	0.741	5.33	-0.924	-0.176	0.567		**√**
**C4-9**	0.872	30.356	0.957	1.03	0.713	4.361	-1.026	-0.181	0.618		**√**
**C4-10**	0.876	28.958	0.957	1.00	0.732	4.171	-1.104	-0.246	0.616		**√**
**C4-11**	0.830	25.185	0.958	1.01	0.691	3.18	-1.207	-0.225	0.598		**√**

*Note* (1) represents correlation coefficient analysis; (2): the critical ratio; (3): Cronbach’s α coefficient analysis; (4): discrete tendency analysis; (5): factor analysis

* represents that the item was deleted, and the bold form means that the item does not meet the criteria

**Table 3:** Assumptions of unidimensionality of the dimensions for the scale

	Dimension	KMO	Bartlett’s Test of Sphericity	The first eigenvalues	The second eigenvalues	Ratio
**Part B**	**B1**	0.881	*p* < 0.001	5.187	0.994	5.22
	**B2**	0.947	*p* < 0.001	7.561	0.502	15.06
	**B3**	0.924	*p* < 0.001	4.803	0.369	13.01
	**B4**	0.948	*p* < 0.001	7.541	0.510	14.79
	**B5**	0.749	*p* < 0.001	2.526	0.278	9.09
**Part C**	**C1**	0.849	*p* < 0.001	3.232	0.360	8.98
	**C2**	0.828	*p* < 0.001	3.202	0.380	8.43
	**C3**	0.864	*p* < 0.001	3.378	0.261	12.94
	**C4**	0.951	*p* < 0.001	7.956	0.736	10.80

Following the extensive screening and refinement process, the MINCS is now organized into three distinct parts: Part A: This section is dedicated to gathering the basic demographic characteristics of the respondents. Part B encompasses 38 elements of missed nursing care in the ICU, categorized into five dimensions. These dimensions include: 5 items related to patients’ physiological needs; 13 items associated with safety needs; 7 items pertaining to emotional needs; 10 items concerning esteem needs; and 3 items addressing cognitive needs. In Part C, there are 25 items that serve as potential reasons for such missed intensive nursing care. These items are grouped into four dimensions, 6 items associated with labor resources;4 items in the domain of material resources; 4 items related to communication factors; and 11 items pertaining to managerial factors. This well-structured framework allows for a comprehensive assessment of missed nursing care in the ICU, covering both the elements of care and the potential reasons behind their omission.

### Results of scale validation

#### Construct validity of the MINCS

The confirmatory factor analysis (CFA) provided valuable insights into the construct validity of the MINCS (Table 4). The overall fit indices for the adjusted model were found to be acceptable in both Part B and Part C of the MINCS: for Part B, *χ*^2^ /df = 4.453, RMSEA = 0.079, SRMR = 0.0496, IFI = 0.913, TLI = 0.904, CFI = 0.912; for Part C: *χ*^2^ /df = 4.374, RMSEA = 0.078, SRMR = 0.0313, IFI = 0.946, TLI = 0.939, CFI = 0.946. In Part C, two items with lower item factor loadings (< 0.6) were removed, further enhancing the scale’s construct validity. Standardized factor loadings were observed to range from 0.659 to 0.917 in Part B and 0.789 to 0.935 in Part C. These results indicate that the final MINCS (Appendix S2) demonstrates favorable construct validity, reinforcing its ability to accurately measure the intended construct.

**Table 4:** Goodness-of-fit statistics of the scale

Indices	Criteria	Part B	Part C
**χ2/df**	< 5	4.453	4.374
**RMSEA**	< 0.08	0.079	0.078
**IFI**	≥ 0.90	0.913	0.946
**TLI**	≥ 0.90	0.904	0.939
**CFI**	≥ 0.90	0.912	0.946
**PGFI**	≥ 0.5	0.655	0.695
**PNFI**	≥ 0.5	0.815	0.824

*Note χ*^2^*/df*: Chi-square value/degree of freedom; *RMSEA*: root mean square error of approximation; *IFI*: Incremental fitting index; *TLI*: Tucker‒Lewis index; *CFI*: Comparative fitting index; *PGFI*: Parsimonious goodness-of-fit; *PNFI*: Parsimonious normed fit index

#### Reliability of the MINCS

Cronbach’s α coefficients were high, ranging from 0.896 to 0.969 for each dimension in Part B and from 0.917 to 0.968 in Part C. The overall MINCS demonstrated strong reliability with a coefficient of 0.951, which was above the threshold of 0.70. The correlation of the two halves of the scale was 0.991 and 0.986 in two parts, showing great split half reliability. The test–retest reliability was 0.96 and 0.97 in Part B and Part C, respectively.

## Discussion

This study aimed to develop and validate a measurement tool for assessing missed nursing care in adult intensive care units. The process adhered to scientific procedures, integrating principles from both classical test theory and item response theory methods. Our rigorous validation and reliability analyses confirmed that the MINCS effectively measures missed care elements and their causes within the ICU, offering a valuable reference for assessing care quality and developing targeted interventions. This study contributes to patient-centered approaches in ICU care and provides a foundation for further research and innovation in improving patient-centered care practices.

Focusing on the principles of patient-centered holistic care [47], our evaluation of nursing activities in ICU settings was rooted in the consideration of whether these activities addressed patients’ needs, as delineated by Maslow’s hierarchy of needs theory. This theory encompasses physiological, safety, belongingness, esteem, and cognitive needs, offering a comprehensive framework for assessing whether ICU nurses deliver holistic care to critically ill patients. Instances of missed nursing care are more likely when these fundamental needs go unmet [48]. The greatest challenge in nursing currently is to provide holistic care to meet all aspects of patients’ needs [49], by selecting Maslow’s hierarchy of needs theory, we can identify which nursing needs of patients have not been met and areas that require further improvement in the caring process. Previous measurement tools predominantly focused on the satisfaction of patients’ physiological needs [3,5 ], often neglecting social and psychological aspects. In contrast, our developed tool evaluates care interventions from various dimensions, including physiological, psychological, and social aspects. Additionally, these tools treated nursing care activities as independent tasks, rarely assessing the structural validity of the missing content [10, 49]. Adhering to our theoretical framework, we identified and extracted five factors in Part B, closely aligning with our expectations. The final factor analysis results demonstrated factor loadings exceeding 0.4 in each dimension, without any cross-loading [34]. It should be noted that the initial dimension of physiological needs comprised 14 items covering nutrition, water, oxygen, excretion, pain, sleep, positioning, rest and activity, and comfort. However, the results of the first round of EFA suggested that item 1 “Ensuring patient airway patency,” item 2 “Providing corresponding care according to different respiratory support methods,” and item 11 “Turning patients according to their condition” should be classified under the safety needs dimension. After discussions with experts, it was agreed that these items are indeed closely associated with patient safety. Therefore, we concurred with this modification. Six items were removed for not meeting factor analysis criteria (loading value > 0.4 or without cross-loadings). Consequently, the physiological needs dimension now comprises only five items, specifically addressing nutrition, thirst, and sleep. When the content within these five dimensions is assessed as missed care, it signifies that the needs of patients in these aspects have not been fulfilled. In terms of exploration of missed care elements, this study can be considered one of the few that identifies MNC from the patient’s perspective [50], making the MINCS tool unique compared to other instruments.

Our investigation into the reasons for missed care led us to categorize them into labor resources, material resources, communication, and managerial factors. Notably, Kalisch’s tool [11], addresses staffing shortages or a surge in patient numbers in the human resources section of the reasons for missed care. In our tool, this category incorporates additional factors derived from interview results. Factors contributing to missed nursing care in the ICU may include both an absolute shortage of nursing staff and a relative lack of competence. The ICU sets a high standard for nursing quality, necessitating nurses with advanced theoretical knowledge and proficiency in utilizing sophisticated medical equipment. Collaboration with multidisciplinary teams, such as partnering with rehabilitation specialists for early patient mobilization, further elevates the expectations placed on ICU nurses. In recent years, the impact of nursing management on missed care has gradually been explored [51], align seamlessly with our qualitative interview results. Consequently, synthesizing these two sources, we identified an additional factor contributing to missed care: managerial factors. This inclusion introduces new dimensions to the understanding of missed care, offering fresh perspectives for the development of intervention measures.

During the scale validation process, confirmatory factor analysis (CFA) supported a five-factor structure in Part B and a four-factor structure in Part C, consistent with previous studies [48, 52]. To gauge the scale’s reliability, we employed various methods, including Cronbach’s α coefficient, split-half reliability, and test-retest reliability. In both Part B and Part C, all coefficients exceeded 0.8, signifying strong internal consistency for the MINCS.

There were several strengths of this study to be underlined. First, we adhered to a rigorous and transparent scale development process, ensuring the precision and practicality of the MINCS. Second, we employed a comprehensive approach by combining CTT and IRT methods to thoroughly assess item quality. Third, the MINCS covers the evaluation of patients’ needs across five dimensions, aligning with the principles of holistic nursing care and patient-centered care. Employing this tool for clinical department nursing quality evaluation facilitates the timely identification of issues and the implementation of corrective measures, ultimately enhancing nursing care outcomes and patient satisfaction. Nonetheless, it is important to acknowledge certain limitations. MINCS requires participants to recall and self-report instances of missed care in their daily work, which can be a sensitive topic. Respondents may be hesitant to disclose the true extent of the issue, potentially raising concerns about the authenticity of the study results. Moreover, the scale encompasses a total of 74 items across its three parts, potentially imposing a response burden on participants. Additionally, as with many survey-based studies, our research focused on understanding missed nursing care through survey responses, thus establishing causality is challenging. In future investigations, these limitations must be taken into consideration.

## Conclusion

In this study, we successfully developed and validated the Missed Intensive Nursing Care Scale (MINCS), a new instrument for assessing missed nursing care in adult intensive care units. The scale demonstrated strong reliability and validity, making it a valuable tool for evaluating nursing quality in ICU patient care. Nurse managers can utilize the results of MINCS assessments to design interventions aimed at enhancing the overall quality of care and ultimately improving patient satisfaction. As we move forward, future research can explore the applicability of MINCS in diverse cultural contexts, further validating and adapting the scale to address nursing care quality across a broader spectrum.

### Electronic supplementary material

Below is the link to the electronic supplementary material.

Supplementary Material 1

Supplementary Material 2

## Data Availability

The datasets used and/or analyzed during the current study are available from the corresponding author on reasonable request.

## Abbreviations

MNC: Missed nursing care
MINCS: Missed intensive nursing care scale
ICU: Intensive care unit
PIRNCA: Perceived implicit rationing of nursing care
CVI: Content validity index
EFA: Exploratory factor analysis
CFA: Confirmatory factor analysis
CTT: Classical test theory
IRT: Item response theory
SD: Standard deviation
RMSEA: Root mean square error of approximation
IFI: Incremental fitting index
TLI: Tucker-Lewis index
CFI: Comparative fitting index
PGFI: Parsimonious goodness-of-fit
PNFI: Parsimonious normed fit index
