# Supplementary material for: Development and validation of the missed intensive nursing care scale
Source: BMC Nurs. 2024 Mar 7;23:165. doi: 10.1186/s12912-024-01805-3 (PMC10919009; doi:10.1186/s12912-024-01805-3)
Supplement: Supplementary file 1 — Supplementary Material 1 [file 12912_2024_1805_MOESM1_ESM.docx]

**Appendix S2**

**Part B Elements of missed nursing care in ICU**

| **Nursing care related to physiological needs** | **B1-1** | Assessing patient nutritional status |
| --- | --- | --- |
|  | **B1-2** | Evaluating the effectiveness of enteral/ parenteral nutrition |
|  | **B1-3** | Assessing patient thirst levels dynamically |
|  | **B1-4** | Implementing individualized thirst management interventions |
|  | **B1-5** | Assessing patient sleep quality and solve sleep-related problem |
| **Nursing care related to safety needs** | **B2-1** | Ensuring patient airway patency (e.g., timely suctioning) |
|  | **B2-2** | Providing corresponding care according to different respiratory support methods (e.g., Non-invasive/Invasive Ventilation) |
|  | **B2-3** | Turning patients according to their condition |
|  | **B2-4** | Strictly implementing patient identification and verification procedures |
|  | **B2-5** | Carefully monitoring vital signs and changes in the patient's condition |
|  | **B2-6** | Following protocols for daily awakening of sedated patients when clinically appropriate |
|  | **B2-7** | Maintaining accurate and comprehensive nursing documentation for each shift |
|  | **B2-8** | Assessing the risk of adverse events in patients (adverse events refer to falls, bed exits, unplanned extubations, etc.) |
|  | **B2-9** | Assessing indications for physical restraints in patients and implementing rational and effective restraint care |
|  | **B2-10** | Implementing preventive nursing measures for avoiding adverse events in patients |
|  | **B2-11** | Executing measures for preventing and controlling hospital-associated infections (HAIs) |
|  | **B2-12** | Maintaining the safety of the medical equipment |
|  | **B2-13** | Ensuring safe patient transfers |
| **Nursing care related to belongingness needs** | **B3-1** | Communicating with family members and encouraging their involvement to harness their positive support role |
|  | **B3-2** | Treating patients with an attitude that is proactive, warm, patient, and friendly |
|  | **B3-3** | Employing diverse and scientifically sound methods to dynamically assess the mental/psychological state of patients |
|  | **B3-4** | Implementing targeted and individualized care measures based on assessment results, including proactive use of both verbal and non-verbal behaviors (such as touch, handshakes) for effective communication with patients |
|  | **B3-5** | Continuously evaluating the effectiveness of communication and interaction |
|  | **B3-6** | Providing stimulatory care for comatose patients (**Stimulatory care** refers to the application of comprehensive sensory stimulation techniques, including sound, light, temperature, acupuncture, massage, etc., apart from surgical or medical interventions for reducing intracranial pressure, improving cerebral blood circulation, and utilizing hyperbaric oxygen therapy to enhance brain cell metabolism in patients with brain injuries, helping them regain consciousness sooner) |
|  | **B3-7** | Creating a patient-centered healthcare atmosphere collectively among department members |
| **Nursing care related to esteem needs** | **B4-1** | Addressing patients with courtesy |
|  | **B4-2** | Understanding the personality traits of patients, and respecting their lifestyle habits/beliefs |
|  | **B4-3** | Before performing nursing procedures, explaining and obtaining informed consent from conscious patients |
|  | **B4-4** | Protecting patient privacy during nursing procedures |
|  | **B4-5** | Inquiring about patients' feelings after procedures and expressing gratitude and appreciation for their cooperation |
|  | **B4-6** | Reasonably applying empathy and compassion in the workplace (**Empathy**, also known as empathy, emotional empathy, emotional resonance, and perspective-taking, refers to the ability to empathize with, understand, and handle the emotions and feelings of others through a process of "putting oneself in someone else's shoes) |
|  | **B4-7** | Actively listening to the opinions of patients and their families |
|  | **B4-8** | Empowering patients to fully participate in clinical diagnosis and nursing decision-making |
|  | **B4-9** | Assisting post-recovery patients with functional exercises |
|  | **B4-10** | Encouraging patients to engage in self-care |
| **Nursing care related to cognitive needs** | **B5-1** | Explaining the necessity and safety of using various medical instruments |
|  | **B5-2** | Daily inform patients of diagnosis, treatment/nursing plans/progress, and their medical condition |
|  | **B5-3** | Daily provide cognitive stimulation training by informing patients of the time, location, people, etc |

**Part C Reasons of missed nursing care in ICU**

| **Labor resources** | **C1-1** | Inadequate competency or core capabilities among nursing staff |
| --- | --- | --- |
|  | **C1-2** | Insufficient sense of responsibility and self-reliance among nursing personnel |
|  | **C1-3** | Occurrence of professional burnout and diminished job satisfaction among nursing staff |
|  | **C1-4** | Limited understanding and low priority placed on patient safety and overall care accountability by nursing personnel |
| **Material resources** | **C2-1** | Insufficient supply of departmental resources such as medications and equipment |
|  | **C2-2** | Outdated and inconvenient-to-use medical equipment and devices within the department |
|  | **C2-3** | Insufficiently intelligent electronic health record systems and health information systems |
|  | **C2-4** | Design flaws in departmental infrastructure, such as handwashing sinks and bed unit usage area |
| **Communication factors** | **C3-1** | Inadequate communication and collaboration among nursing teams (e.g., insufficient shift handovers |
|  | **C3-2** | Inadequate communication and collaboration between medical and nursing staff and patients/families |
|  | **C3-3** | Inadequate communication and collaboration among medical and nursing staff |
|  | **C3-4** | Inadequate communication and collaboration with other departments/auxiliary personnel (e.g., pharmacy, logistics staff) |
| **Managerial factors** | **C4-1** | Unreasonable allocation of nursing labor resources |
|  | **C4-2** | Unreasonable scheduling |
|  | **C4-3** | Insufficient training and assessment for nursing staff |
|  | **C4-4** | Insufficient emotional or financial support from management for nursing staff |
|  | **C4-5** | Frequent occurrences of nursing interruptions events **(Nursing interruption events** refer to the process of providing nursing services to patients in defined time, roles, and treatment settings, which are interrupted by unforeseen events or events that distract attention) |
|  | **C4-6** | Unreasonable department performance management system |
|  | **C4-7** | Lack of supervision for inappropriate nursing behavior |
|  | **C4-8** | Lack of reasonable, effective, and standardized nursing processes |
|  | **C4-9** | Rigid nursing models (e.g., failure to implement a patient-centered nursing model) |
|  | **C4-10** | Lack of a humanistic care philosophy in the department |
|  | **C4-11** | Inappropriate ward layout |
