# Supplementary material for: Development and validation of the missed intensive nursing care scale
Source: BMC Nurs. 2024 Mar 7;23:165. doi: 10.1186/s12912-024-01805-3 (PMC10919009; doi:10.1186/s12912-024-01805-3)
Supplement: Supplementary file 2 — Supplementary Material 2 [file 12912_2024_1805_MOESM2_ESM.docx]

**Interview guides**

1. How do you perceive the physiological/safety/belongningness/esteem/self-actualization/cognitive/aesthetic needs of ICU patients? Besides these needs, are there any other needs for them?

2. As nurses, what can we do to meet those needs? And to what extent do you feel that the needs are being met?

3. Do you think any missed nursing care occurs during the process of needs satisfaction? Why?

If yes, what measures do you think can be taken to prevent or reduce the occurrence of this problem?
